# Targeting Senescence in Oncology: An Emerging Therapeutic Avenue for Cancer

**DOI:** 10.3390/curroncol32080467

**Published:** 2025-08-18

**Authors:** Satoru Meguro, Syunta Makabe, Kei Yaginuma, Akifumi Onagi, Ryo Tanji, Kanako Matsuoka, Seiji Hoshi, Tomoyuki Koguchi, Emina Kayama, Junya Hata, Yuichi Sato, Hidenori Akaihata, Masao Kataoka, Soichiro Ogawa, Motohide Uemura, Yoshiyuki Kojima

**Affiliations:** Department of Urology, School of Medicine, Fukushima Medical University, 1 Hikarigaoka, Fukushima 960-1295, Japan; smakabe@fmu.ac.jp (S.M.); uro-yagi@fmu.ac.jp (K.Y.); onagi@fmu.ac.jp (A.O.); tanji422@fmu.ac.jp (R.T.); kanaco@fmu.ac.jp (K.M.); uro-hosi@fmu.ac.jp (S.H.); gucchii@fmu.ac.jp (T.K.); emina@fmu.ac.jp (E.K.); akju826@fmu.ac.jp (J.H.); ysato@fmu.ac.jp (Y.S.); hakai@fmu.ac.jp (H.A.); masaoka@fmu.ac.jp (M.K.); soh@fmu.ac.jp (S.O.); muemura@fmu.ac.jp (M.U.); ykojima@fmu.ac.jp (Y.K.)

**Keywords:** cellular senescence, cancer, tumor microenvironment, therapy-induced senescence, senotherapeutics

## Abstract

Since cancer is often linked to the aging process, the importance of cellular senescence in cancer has come under the spotlight. While senescence in cancer cells can serve as a natural barrier against cancer due to its proliferation arrest, its secretory phenotypes and alterations in the surface proteome can paradoxically promote or suppress tumor progression. Senescent cancer-associated fibroblasts, endothelial cells, and immune cells can also contribute to cancer promotion. During therapeutic interventions for cancer, not only their therapeutic effects, but also therapy-induced senescence may have an impact on cancer outcomes. Senotherapeutics, therapy targeting senescent cells, have been reported as novel cancer therapy in recent studies, and the combination of senescence induction and senotherapeutics has been increasingly recognized. Although some clinical trials of senotherapeutic drugs for cancer with or without senescence-inducible therapy are ongoing, there is as yet no satisfactory clinical application. With further research into targeting senescence in oncology, it is expected that senotherapeutics, particularly in combination with senescence-inducing therapy, will become a novel therapeutic strategy.

## Simple Summary

Cellular senescence has a double-edged role in cancer, including suppressing and promoting cancer development. While senescence in cancer cells inhibits their growth, its secretory phenotype and surface proteome alterations can paradoxically promote or suppress tumor progression. Senescence in stroma or immune cells can also promote cancer. Senotherapeutics, targeting senescent cells, have been focused on as a novel strategy of cancer therapy; specifically, the combination of senescence-inducing treatments and senotherapeutics can improve therapeutic efficacy. Because effective clinical application remains limited, further research is needed to facilitate the use of senotherapeutics as a novel therapeutic approach in oncology.

## 1. Introduction

Cellular senescence is a stress response program in cells, defined as permanent cell cycle arrest. With physiological and pathological stresses, such as DNA damage, telomere dysfunction, oncogene activation, and organelle stress, normal cells turn into senescent cells [1,2]. Senescent cells have specific intracellular metabolic pathways, and even an extracellular effect on bystander cells and accumulated senescent cells in tissue and organs leads to changes in tissue homeostasis. In this context, cellular senescence has been reported to be associated with several diseases, in particular, age-related diseases [3,4]. Many studies in the oncology field have indicated that cellular senescence is relevant to several types of cancer as age-related diseases, and senescent cells have recently been identified as a novel drug therapy target.

The role of cellular senescence in cancer is heterogeneous, as senescent cells can paradoxically exert both tumor-promoting and tumor-suppressive effects. Whether senescent cells act as a “friend or foe” in cancer likely depends on factors such as the organ and tissue context, the cell type undergoing senescence, and the nature of the senescence-inducing stimulus. In a tumor microenvironment, cellular senescence occurs not only in cancer cells but also in stroma and immune cells. These senescent cells exhibit diverse alterations in their internal metabolism, surface proteome, and secretory phenotype, allowing them to communicate with both senescent and non-senescent cells. Through these interactions, they contribute to the formation of a complex cellular network that influences tumor fate [2,4].

In this review, the relevance of cellular senescence in cancer and advanced cancer therapies targeting senescent cells are presented, with reference to some of the latest research.

## 2. Hallmarks of Cellular Senescence

### 2.1. General Features of Senescent Cells

Cellular senescence has traditionally been characterized as a cellular state marked by irreversible cell cycle arrest in response to internal or external stresses. This phenomenon was first reported in cultured cells and described as cellular senescence in 1961 by Hayflick and Moorhead. They reported that cultured human normal fibroblasts presented a finite proliferative capacity, after which the cells halted dividing and eventually entered a senescence state: they termed this state “the Phase III”, when cell replication stopped but remained metabolically active [5]. Cyclin-dependent kinase (CDK) inhibitors 1A and 2A (known as p21^Waf1/Cip1^ and p16^INK4a^, respectively) are the key regulators of stable cell cycle arrest, and their expressions are increased in senescent cells [6]. Accumulated p16 and p21 hinder CDK2 and CDK4/6 activity, leading to decreased phosphorylation of retinoblastoma (Rb). In this process, Rb does not detach from transcription factor E2F, inhibiting the E2F function of driving the cell cycle [7]. Moreover, senescent cells are also characterized by their resistance to apoptosis caused by upregulated BCL-2 family proteins [8,9], the elevated lysosomal enzyme of senescence-associated β galactosidase (SA-β-gal) activity [10], the loss of lamin-B1 [11], defective mitochondria, and deregulated metabolism [12]. As structural features, senescent cells show some morphological changes, including enlarged cell and nuclear size, an abnormal nuclear envelope, and irregular chromosome condensation and distribution [13].

### 2.2. Senescence-Associated Secretory Phenotype

The senescence-associated secretory phenotype (SASP) is the robust transcriptional activation for paracrine effects triggered by cellular senescence. Senescent cells display their pleiotropic biological functions in their tissue microenvironment via the SASP, marked by the secretion of cytokines, chemokines, growth factors, and proteases [2]. The SASP is regulated by several pathways, such as the p53-p21 and p16-Rb pathways, the NF-κB pathway, the p38 MAPK and mTOR pathways, and the cytosolic DNA-cGAS-STING pathway [14]. Representative SASP factors include proinflammatory interleukins (e.g., IL-1α, IL-1β, IL-6, and IL-8) [15,16,17], chemokines (e.g., C-C motif ligand (CCL) 2, C-X-C motif ligand (CXCL) 1, CXCL2, CXCL5, CXCL12) [18,19,20,21,22,23], and growth factors (e.g., TGFβ and GDF15) [24,25,26,27]. SASP factors serve as key paracrine signals mediating communication between senescent cells and neighboring cells, such as stromal cells, immune cells, and both premalignant and malignant cells. Moreover, the SASP induces senescence in neighboring non-senescent cells in a paracrine manner [28]. Notably, differences in the composition and levels of the SASP are observed across various tissues and models of senescence. Senescent cell phenotypes even change over time; thus, cells in the early stages of senescence differ from those in the advanced stages [29]. In this context, the SASP presents both advantages and disadvantages for cancer development in different physiological and pathological states and temporal phases.

## 3. Senescence Suppresses Cancer

### 3.1. Tumor Cell Growth Arrest

**Figure 1:**
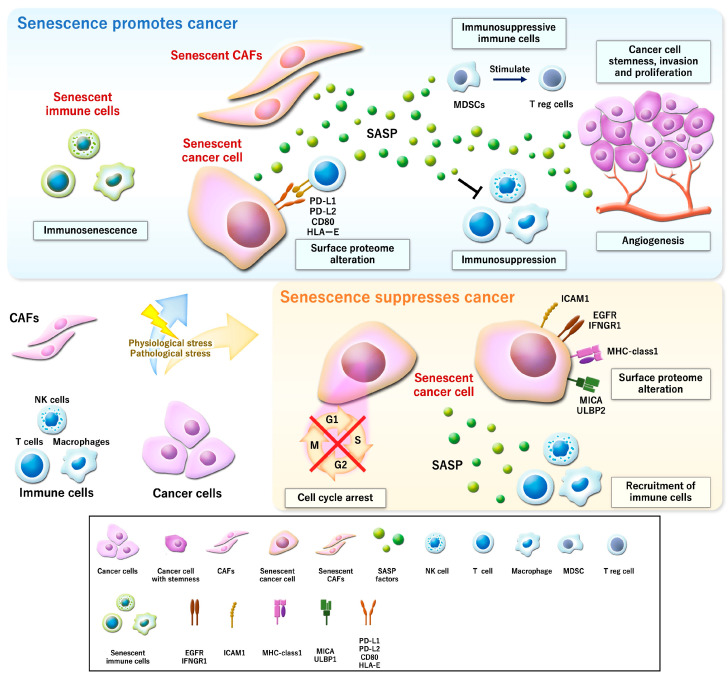
Senescence promotes or suppresses cancer. In a tumor microenvironment, cellular senescence can occur in cancer cells, stroma, and immune cells via physiological or pathological stress. While senescence in cancer cells commonly exhibits tumor-suppressive effects via cell cycle arrest, the recruitment of immune cells, and surface proteome alteration, senescence can paradoxically contribute to tumor growth through several mechanisms that create an immune-suppressive and cancer-favorable microenvironment. The figures were created by Adobe Illustrator and Photoshop. CAFs, cancer-associated fibroblasts; SASP, senescence-associated secretory phenotype; NK cells, natural killer cells; MDSCs, myeloid-derived suppressor cells; T reg cells, regulatory T cells; PD-L1/2, programmed death ligand 1/2; ICAM1, intercellular adhesion molecule 1; EGFR, epidermal growth factor receptor; IFNGR1, interferon gamma receptor 1; MHC-class1, major histocompatibility complex class 1; MICA, MHC class I polypeptide-related sequence A; ULBP2, UL16 binding protein 2; HLA-E, human leukocyte antigen-E.

One definite function of cellular senescence is to prevent tumor development (Figure 1). Due to the permanent autonomous cell cycle arrest via p53, p16, and p21, the definitive feature of cellular senescence, cellular senescence serves as a natural defense mechanism preventing tumorigenesis. In particular, oncogene-induced senescence (OIS) acts as a safeguard against tumorigenesis. Whereas certain aberrant signaling pathways and dysregulated DNA replication enhance proliferative signals, they paradoxically reinforce irreversible cell cycle arrest, thereby preventing malignant transformation [30]. This research reported that senescence mediated by the methylation of histone H3 lysine 9 (H3K9me), induced by the acute induction of oncogenic RAS and involving Rb signaling, was dependent on histone methyltransferase Suv39h1, and Suv39h1-deficient mice showed the aggressive formation of lymphoma, as the silencing of growth-promoting genes was impaired. OIS is frequently associated with the activation of key signaling cascades, including the BRAF–MEK–ERK and PI3K–AKT–mTOR pathways [31]. Moreover, OIS is also triggered by the inactivation of tumor suppressor genes, including PTEN and NF1 [32]. Interestingly, it has recently been reported that even the level of oncogenic stress determined the cell fate between senescence and a tumor-initiating phenotype [33].

### 3.2. Recruitment of Immune Cells

Mediated by inflammatory SASP components, a cellular senescence program has been reported to be relevant to tumor suppression by recruiting diverse populations of immune cells such as natural killer (NK) cells, neutrophils, macrophages, and T cells [2] (Figure 1). For example, the p21-regulated SASP altered immunosurveillance including macrophage activity via CXCL14 secretion to eliminate premalignant cells. Interestingly, this research indicated a “timer” mechanism: cells which presented a p21-activated secretory phenotype attracted macrophages via CXCL14. Attracted macrophages monitored these stressed cells and disengaged from the cells if p21 was normalized within 4 days. Otherwise, macrophages recruited cytotoxic T cells that eliminated cells [34]. Oncogene-induced or p53-reactivated senescent cells showed the SASP and were subjected to immune cell clearance, leading to a limitation of tumorigenesis in the liver [35,36]. In another study, upregulated p53 contributed to tumor suppression not only through cell-intrinsic mechanisms, but also by shaping an antitumor microenvironment via secreted factors that modulated macrophage polarization and activity [37]. The SASP induced by combining a mitogen-activated protein kinase (MEK) inhibitor with a CDK4/6 inhibitor promoted the elimination of cancer cells by NK cells and CD8+ T cells in Kras-mutant lung and pancreatic cancer models [38,39]. Moreover, it has recently been reported that cellular senescence in prostate cancer induced by the activation of IL6ST signaling or a retinoic acid receptor agonist increased the transition from an immune-cold to an immune-hot tumor [40,41]. These reports imply that the inflammatory SASP could play a critical role in enabling immune surveillance during senescence.

### 3.3. Alteration of the Surface Proteome

In addition to delivering remodeling signals that affect surrounding tissue through the SASP, senescent cells can enhance their sensitivity to environmental signals by altering the surface proteome [42] (Figure 1). For example, a CDK4/6 inhibitor, a senescence inducer, enhanced antitumor immunity by increasing the tumor antigen presentation in mouse models of breast carcinoma and other solid tumors. This research identified two mechanisms by which a CDK4/6 inhibitor enhances the antitumor immune response. First, a CDK4/6 inhibitor activated the expression of endogenous retroviral elements in tumor cells and increased intracellular levels of double-strand DNA. This stimulated type 3 interferon production and facilitated further tumor antigen presentation. Second, a CDK4/6 inhibitor suppressed regulatory T cell proliferation [43]. Another study reported that not only major histocompatibility complex class 1 (MHC-class 1), but also various growth factors and cytokine receptors such as epidermal growth factor receptor (EGFR), intercellular adhesion molecule 1 (ICAM1), and interferon gamma receptor 1 (IFNGR1), were increased on senescent cells [44]. Moreover, ligands of NK cells including MHC class I polypeptide-related sequence A (MICA) and UL16 binding protein 2 (ULBP2) were upregulated in senescent cells, dependent on initial DNA damage and the ERK signaling pathway [45]. These studies indicate that not only external signals from senescent cells, but also their internal changes can modulate cancer fate.

## 4. Senescence Promotes Cancer

### 4.1. SASP-Driven Cancer Promotion

Although senescence is typically a tumor-suppressive process, cell-extrinsic factors such as the SASP can paradoxically support tumor development (Figure 1). Though various immune populations contributing to the elimination of cancer are recruited into the tumor microenvironment by the SASP, attenuation of the host immune system against the malignant population can also occur. IL-6 secreted from senescent cells was reported to attract myeloid-derived suppressor cells (MDSCs), allowing an immunosuppressive environment that cannot overcome cancer promotion [46]. Another study also reported that C-C motif chemokine receptor type 2 (CCR2^+^) MDSCs recruited by CCL2 from senescent hepatocytes inhibited NK cell-driven immune monitoring [47]. The accumulated MDSCs stimulated the function of immunosuppressive regulatory T cells [48], leading to the further negative modulation of antitumor immunity. In prostate cancer lacking PTEN, activated JAK2/STAT3 signaling established an immunosuppressive tumor microenvironment via SASP factors such as CXCL1, CXCL2, and IL-6 and resulted in tumor growth and chemoresistance [49].

Other than SASP-mediated immunosuppression, the SASP can promote tumorigenesis through several mechanisms. IL-6 has also been reported to be relevant to tumorigenesis, stemness in tumor cells, angiogenesis, and metastasis [50,51,52]. IL8 enhanced the mitigation ability and stemness in breast cancer cells [50]. CCL5 from senescent cells facilitated the cell proliferation, invasion, mitigation, and angiogenesis of prostate hyperplasia cells or non-small-cell lung cancer cells [53,54]. Matrix metalloproteinases (MMPs), a family of enzymes that breaks down components of the extracellular matrix, have been reported as representative SASP factors. MMP-3 and MCP-1 enhanced breast cancer growth and proliferation [55]. Another study indicated that MMPs as reprogrammed SASP induced by TIMP deletion fostered the metastasis of PTEN-null prostate cancer [56]. Soluble E-cadherin secreted from senescent cells facilitated melanoma cell metastasis and invasion [57]. Moreover, CXCL12 from senescent thyroid cancer cells contributed to cancer invasion and metastasis to lymph nodes [58].

### 4.2. Ligands on Senescent Cells to Evade Immune Attack

Similar to typical tumor cells, senescent cells have been reported to be able to evade immune surveillance by expressing immune checkpoint ligands [59] (Figure 1). Senescent cells upregulated PD-L1 and facilitated an immunosuppressive milieu that could promote the accumulation of senescent cells, possibly affecting cancer progression [60,61,62]. Transforming growth factor β-mediated senescent lymphoma cells exhibited the high-level expression of PD-L1, inhibiting effective removal by host immunity [63]. Breast cancer with senescence-like features after chemotherapy showed increased PD-L1 and CD80 [64]. Senescent cells also can evade immune monitoring with upregulated programmed death-ligand 2 (PD-L2) and recruit immunosuppressive cells, leading to chemotherapy resistance and tumor progression [65]. Moreover, senescent cells can escape immune attack by NK cells and CD8^+^ T cells by expressing human leukocyte antigen-E (HLA-E) [66]. Interestingly, the shedding of (natural killer group 2D) NKG2D ligands on some senescent cells induced by genomic stress was reported to be associated with immune evasion [67].

### 4.3. Senescence in Stroma: Senescent Cancer-Associated Fibroblasts and Senescent Endothelial Cells

Cancer-associated fibroblasts (CAFs) are among the central stromal components of the tumor microenvironment of solid tumors, and it has been suggested that a subpopulation of them can promote cancer progression [68]. In recent years, increasing attention has been directed to the effects of cellular senescence on CAFs in the tumor microenvironment [3]. For example, in pancreatic ductal adenocarcinoma (PDAC), senescent CAFs were reported to drive tumor fibrosis, promote immunosuppressive macrophage phenotypes, and impair T cell function, collectively contributing to disease progression [69,70,71]. In bladder cancer, senescent CAFs secreting CXCL12 can promote cancer progression in mice and humans [18]. Senescent CAFs in breast cancer have been shown to promote tumor progression by altering the extracellular matrix in a way that suppressed natural killer (NK) cell activity, and their presence has been associated with an increased risk of recurrence and worse clinical outcomes in patients [72].

Endothelial cells, which make up vasculatures, are also critical stromal components of a tumor microenvironment. In addition to managing blood flow and vascular permeability, endothelial cells are crucial for the tumor metastasis process, tumor-associated angiogenesis, and immune responses against cancer [73]. Therefore, the alteration of endothelial cell phenotypes by cellular senescence can impact various aspects of the tumor microenvironment and influence cancer progression. Senescent endothelial cells induced by KLF4 have been reported to be enriched in the metastatic tumors of uveal melanoma in the liver and to contribute to tumor cell migration and metastasis formation through SASP [74]. Senescent endothelial cells secreted CXCL11 and increased breast cancer cell proliferation, migration, and invasion in vitro [75]. Sunitinib, a receptor tyrosine kinase inhibitor that targets multiple receptors including vascular endothelial growth factor receptors, has been shown to promote breast cancer progression by inducing senescence and SASP in endothelial cells [76]. Moreover, the sustained activation of Notch1 in endothelial cells induced senescence and a pro-inflammatory phenotype that promoted neutrophil infiltration, tumor cell adhesion, and metastasis [77].

These studies indicate that senescent stroma including senescent CAFs and senescent endothelial cells can be relevant to cancer progression beyond the types of cancer, and may thereby be a novel therapeutic target, as well as senescent cancer cells.

### 4.4. Immunosenescence

Cellular senescence can also occur in immune populations, which is called “immunosenescence” (Figure 1). Immunosenescence causes innate and adaptive immune dysfunction and links to age-related diseases including cancer. Immunosenescence is characterized by thymic atrophy, an imbalance between naïve and memory T cells, metabolic dysregulation, and epigenetic modifications, and similarly shows the SASP developing a pro-inflammatory environment [78]. Immunosenescence is caused not only by natural aging, but also by several environmental factors such as ultraviolet radiation exposure, alcohol, smoking, and pollution [79]. Chronic antigen stimulation is also a factor in immunosenescence [78]. Moreover, even genotoxic cancer therapy can induce immunosenescence. For example, p16-positive senescent T cells were increased in breast cancer patients with chemotherapeutic agents [80]. Elevated *p21* expression was observed after radiation in the white blood cells of patients with hematological diseases [81]. These reports imply that cancer therapy may paradoxically cause cancer progression, mediated by senescence.

## 5. Therapy-Induced Senescence

During the process of cancer treatment, therapy-induced senescence (TIS) is an essential mechanism that affects therapeutic outcomes [42]. TIS represents a more rapid form of cellular senescence triggered by genotoxic stress. As detailed mechanisms, in response to DNA damage, ataxia telangiectasia–mutated (ATM) and ataxia telangiectasia and Rad3-related (ATR) primarily phosphorylate the cell cycle checkpoint kinases Chk1 and Chk2, which subsequently promote the activation of various cyclin-dependent kinase (CDK) inhibitors, and, consequently, these activated CDK inhibitors induce irreversible cell cycle arrest [82,83]. Of note, TIS is induced by not only DNA damage-inducing therapies such as chemotherapy and radiation therapy, but also by molecular targeted therapies such as pan-histone deacetylase (HDAC) inhibitors, a DNA demethylating reagent, MEK inhibitors, CDK inhibitors, EGFR inhibitors, and vascular endothelial growth factor (VEGF) inhibitors [84,85,86,87]. Interestingly, though ATR inhibits the cell cycle in this signaling pathway, the pharmacological inhibition of ATR has conversely been reported to induce cellular senescence [88,89,90].

TIS can contribute to cancer regression. Radiation-induced senescence triggers the SASP, which can help prevent cancer development through systemic immunosurveillance including the activation of NKT cells or M1 macrophage polarization [91,92]. In other research, radiation with a poly (ADP-ribose) polymerase (PARP) inhibitor induced senescence in tumor cells, expressing immunostimulatory cytokines that enhanced the activation of cytotoxic T lymphocyte or dendritic cells (DCs) and promoted a robust antitumor immune response [93,94]. In terms of chemotherapy, doxorubicin has been broadly reported to induce TIS and activate NK cells, T cells, or DCs in multiple myeloma, melanoma, and metastatic breast cancer [95,96,97]. Cisplatin with irinotecan also induced senescence and the SASP, activating T cells and DCs in ovarian cancer, and thereby sensitizing tumors to immune checkpoint inhibitors [98]. Moreover, combination treatment with palbociclib and trametinib has been shown to induce TIS and activate immune surveillance depending on the SASP, ultimately resulting in the inhibition of lung cancer and PDAC promotion [38,39].

However, there have been several reports that TIS promoted cancer progression. In recent research, radiation-induced senescence increased PD-L1 on melanoma cells via glycosylation and decreased T-cell susceptibility, leading to cancer progression [99]. In long-term TIS, a pro-inflammatory and immunosuppressive microenvironment can be created, leading to drug resistance and tumor promotion in breast cancer [100]. SASP factors arising from cisplatin-induced senescence stimulated the proliferation of melanoma cells by activating the ERK1/2-RSK1 signaling pathway [101]. Moreover, doxorubicin-induced senescence in breast cancer with wild-type p53 inhibited the drug response and stimulated cell proliferation and tumor relapse [102].

The impact of TIS on cancer progression is heterogeneous. Though TIS may paradoxically cause unexpected cancer promotion, TIS can contribute positively to cancer treatment by acting synergistically with the original therapeutic regimen. The complicated process of senescence in cancer therapy may play a pivotal role in determining long-term therapeutic outcomes in oncology.

## 6. Senescence-Targeting Therapy in Cancer

### 6.1. Senotherapeutics: Senolytics and Senomorphics

**Figure 2:**
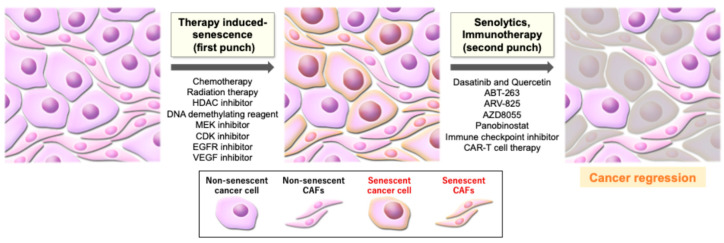
“One-two punch” strategy upon therapy-induced senescence.

Given the cohesive evidence that cellular senescence is involved in the promotion of various types of cancer, senotherapeutics, including senolytics and senomorphics, are expected to become a novel strategy for cancer therapy. Senolytics work by eliminating senescent cells, thereby mitigating the impact of cellular senescence on cancer promotion. Leveraging their ability to eliminate senescent cells, senolytics are used not only as monotherapy, but they are also frequently combined with senescence-inducing treatments such as radiation, chemotherapy, or other agents in a strategy known as “one-two punch therapy” [103] (Figure 2). The combination of dasatinib and quercetin is the first reported senolytic drug therapy, targeting SRC kinase and PI3K-AKT signaling [103,104]. In this research, a single dose of them improved cardiac function, carotid vascular reactivity, and exercise function in aged mice, and regular administration of them extended the health span in progeroid Ercc1 mice, delaying age-related symptoms, osteoporosis, and the loss of intervertebral disk proteoglycans [104]. This combination therapy has been demonstrated to suppress hepatocellular carcinoma progression in a premature aging mouse model [105], and ovarian cancer metastasis in mice when used in conjunction with carboplatin, a platinum-based chemotherapy drug, or with olaparib, a PARP inhibitor [106]. During almost the same period, ABT-263 (navitoclax), a BCL-2 family inhibitor that targets BCL-2, BCL-XL, and BCL-W, was reported to act as a senolytic drug. This research identified that ABT-263 selectively eliminated senescent cells in vitro across various cell types and species by inducing apoptosis, and the oral administration of ABT-263 to either sub lethally irradiated or normally aged mice effectively eliminated senescent cells [8]. As a senolytic drug, ABT-263 has been shown to inhibit the progression of lung and breast cancers in xenograft mouse models following therapy-induced senescence [107] and ovarian and breast cancers when combined with a PARP inhibitor [108]. Other studies have demonstrated that the ABT-263-mediated elimination of senescent cells in the bladder suppressed the progression of bladder cancer in an orthotopic allograft mouse model [18], and the galacto-conjugation of navitoclax after cisplatin-induced senescence inhibited tumor growth in a human lung cancer xenograft mouse model [109]. An in vivo study showed that AZD8055, a mammalian target of the rapamycin (mTOR) inhibitor, possessed senolytic activity and suppressed liver cancer after senescence was triggered by a CDC7 inhibitor that disrupted DNA replication [110]. ARV-825, a BRD4 inhibitor with senolytic properties, was shown to delay the development of liver cancer in mice [111]. A histone deacetylase inhibitor, Panobinostat, was also shown to suppress non-small cell lung cancer and head and neck squamous cell carcinoma cell lines in conjunction with chemotherapy that induced senescence [112]. Moreover, the inhibition of glutaminase 1, that was essential for senescent cell survival, was shown to eliminate senescent cells, and the inhibitor drug could be a novel senolytic strategy [113]. However, although several kinds of senolytics have been reported so far, most of them have only been identified to be effective in a limited number of specific cancer types and also there has not been much evidence regarding the effects of some senolytics including dasatinib + quercetin or panabinostat. On the contrary, some studies have indicated that dasatinib + quercetin did not show the efficacy in mouse liver cancer [114,115] and that panabinostat had no prominent anticancer effect in human refractory renal carcinoma and human castration-resistant prostate cancer [116,117]. While the utility of ABT-236 as senolytics is becoming relatively established, universal senolytics effective across a broad range of cancers have not been identified to date.

Senomorphics can suppress aging-associated phenotypes without eliminating senescent cells. For example, blocking the stromal p38MAPK/MK2 pathway was found to decrease breast cancer metastasis, reduce therapy-related bone loss, and extend survival in mouse models [118]. Moreover, an mTOR inhibitor, rapamycin, attenuated the inflammatory components of the SASP and decreased several cytokines, suppressing prostate cancer growth in mice [15].

Senolytics and immunotherapy eliminate senescent cells and are often combined with senescence-inducing treatments in a strategy known as “one-two punch” therapy. The figures were created by Adobe Illustrator and Photoshop.

CAF, cancer-associated fibroblast; HDAC inhibitor, histone deacetylase inhibitor; MEK inhibitor, mitogen-activated protein kinase inhibitor; CDK inhibitor, cyclin-dependent kinase inhibitor; EGFR inhibitor, epidermal growth factor receptor inhibitor; VEGF inhibitor, vascular endothelial growth factor inhibitor; CAR-T cell therapy, chimeric antigen receptor T cell therapy.

### 6.2. Immune-Dependent Clearance of Senescent Cells

In the context of one-two punch therapy, immunotherapeutic reagents can also act as senolytics as the “second punch” after the “first punch” of TIS. As previously mentioned, senescent cells can recruit immune cells via the SASP and alter the surface proteome associated with immunosurveillance. This modified environment could make immunotherapy more effective against tumor cells. For example, CDK4/6 inhibition induced senescence features in malignant cells and exhibited a synergistic therapeutic effect in melanoma tumors when combined with immunotherapy, overcoming therapy resistance [119]. Another study also reported that a combination of MEK and CDK4/6 inhibitors with an immune checkpoint blockade suppressed PDAC proliferation through the induction of senescence and the SASP modulating tumor vasculature and the immune system [38]. In addition, following senescence induction by MEK and CDK4/6 inhibitors, senolytic CAR-T cell therapy targeting the urokinase-type plasminogen activator receptor was reported to selectively ablate senescent cells and extended the survival of mice with lung adenocarcinoma [120].

### 6.3. Perspectives on Clinical Applications

Although many preclinical studies have presented cohesive evidence suggesting the effectiveness of senotherapeutics for cancer, there have been no clinical applications so far. Nevertheless, several clinical trials of senolytics targeting cancer are ongoing (http://clinicaltrials.gov). Trials testing dasatinib and quercetin with chemotherapy or immunotherapy for breast cancer or head and neck squamous cell carcinomas are underway, and a phase 2 trial testing dasatinib and quercetin with CAR-T therapy for relapsed or refractory multiple myeloma will start soon (Table 1). In terms of ABT-263, over 20 trials of ABT-263 for various types of cancer are active or completed. Of them, some trials combined senescence-inducible therapy with ABT-263, implying its effectiveness as a senolytic drug for cancer following TIS (Table 2). In these trials, although ABT-263 has been generally tolerated, adverse events such as thrombocytopenia, neutropenia, anemia, diarrhea, nausea, vomiting, and decreased appetite have been observed. Similarly, of the active or completed trials of Panobinostat for cancer, several trials have tested its effectiveness for cancer combined with senescence-inducible therapy (Table 3). The main adverse events have included thrombocytopenia, neutropenia, and fatigue. These trials of ABT-263 and Panobinostat have partly resulted in a better response; however, they are relatively old trials and further optimization of dose or schedule is still needed. Together, although senotherapeutics have potential as a strategy for cancer therapy, their effect on cancer in clinical settings has not been remarkable so far. Further in-depth studies seem to be needed to fully leverage the potential of senotherapeutics.

## 7. Conclusions

Given that cancer is often regarded as an age-related disease, targeting cellular senescence in the field of oncology may be important in cancer research. To date, many preclinical studies have addressed cellular senescence in cancer. Moreover, several clinical trials of combining therapies in which senescence induction and senolytics may produce synergy are underway and may be a promising new strategy for cancer. However, because the characteristics of cellular senescence vary depending on senescence inducers, organs, tissues, cancer types, and even cell types, the impact of cellular senescence on cancer is heterogeneous, and it cannot be said that senotherapeutics are always beneficial for cancer therapy. Therefore, to achieve the benefits of senotherapeutics in clinical practice, further detailed research is needed to enable their application to individual patients.

## Figures and Tables

**Table 1:** Clinical trials of dasatinib and quercetin in “one-two punch” strategy for cancer. CAR-T cell therapy, chimeric antigen receptor T cell therapy.

Identifier	Phase	Type of Cancer	Senescence Inducer (First Punch)	Senolytics (Second Punch)	Status
NCT06355037	2	Triple negative breast cancer	Taxane, Anthracycline, Eribulin, Mesylate, Vinorelbine, Capecitabine, Carboplatin, UTD1, Platinum	Dasatinib and Quercetin	Recruiting
NCT05724329	2	Head and neck squamous carcinomas	-	Dasatinib and Quercetin, Immune checkpoint inhibitor (Tislelizumab)	Active
NCT06940297	2	Elapsed or refractory multiple myeloma	Cyclophosphamide, Fludarabine	Dasatinib and Quercetin, CAR-T therapy	Not yet recruiting

**Table 2:** Clinical trials of ABT-263 in “one-two punch” strategy for cancer.

Identifier	Phase	Type of Cancer	Senescence Inducer (First Punch)	Senolytics (Second Punch)	Status	Reference	Adverse Events	Antitumor Effect
NCT05455294	1	Acute myeloid leukemia, myeloid malignancy, myeloproliferative neoplasm	Decitabine	ABT-263	Active			
NCT05222984	1	Recurrent, refractory acute myeloid leukemia	Decitabine	ABT-263	Active			
NCT05192889	1, 2	Refractory, relapsed acute lymphoblastic leukemia	Vincristine, Calaspargase Pegol, Cytarabine, Methotrexate, Mercaptopurine, Cyclophosphamide, Etoposide, Pegaspargase, Erwinia Asparaginase	ABT-263	Active			
NCT03181126	1	Acute lymphoblastic leukemia (ALL), lymphoblastic lymphoma	Vincristine, Pegaspargase	ABT-263	Completed			
NCT02143401	1	metastatic, recurrent malignant solid neoplasm, recurrent hepatocellular carcinoma, refractory malignant neoplasm, Stage IV hepatocellular carcinoma AJCC v7, unresectable solid neoplasm	Sorafenib	ABT-263	Completed			
NCT02079740	1, 2	Metastatic, refractory, unresectable malignant solid neoplasm	Trametinib	ABT-263	Active			
NCT01989585	1, 2	Clinical Stage III, IV cutaneous melanoma AJCC v8, malignant solid neoplasm, metastatic, unresectable melanoma	Dabrafenib, Trametinib	ABT-263	Active			
NCT01009073	1	Solid tumors	Erlotinib, Irinotecan	ABT-263	Completed	[121]	Diarrhea, Nausea, Vomiting, Decreased appetite	27% of disease control rate
NCT00891605	1	Solid tumors	Paclitaxel	ABT-263	Completed	[122]	Alopecia, Anemia, Nausea, Constipation, Diarrhea, Fatigue, Neutropenia, Thrombocytopenia, Vomiting, Decreased appetite, Dehydration, Hypomagnesaemia	Modest antitumor activity
NCT00887757	1	Solid tumors	Gemcitabine	ABT-263	Completed	[123]	Hematologic abnormalities (thrombocytopenia, neutropenia, and anemia), Liver enzyme elevations (ALT and AST), Gastrointestinal disturbances (diarrhea, nausea, and vomiting)	54% of stable disease
NCT00888108	1	Solid tumors	Docetaxel	ABT-263	Completed	[124]	Thrombocytopenia, Fatigue, Nausea, Neutropenia	10% of partial responses
NCT00878449	1	Solid tumors	Etoposide, Cisplatin	ABT-263	Completed	[125]		
NCT00868413	1	Chronic lymphocytic leukemia	Fludarabine/Cyclophosphamide/Rituximab, Bendamustine/Rituximab	ABT-263	Completed			

**Table 3:** Clinical trials of Panobinostat in ’one-two punch’ strategy for cancer.

Identifier	Phase	Type of Cancer	Senescence Inducer (First Punch)	Senolytics (Second Punch)	Status	Reference	Adverse Events	Antitumor Effect
NCT01321346	1	Childhood lymphoblastic/myelogenous leukemia, Hodgkin’s disease, Non-Hodgkin’s disease	Cytarabine	Panobinostat	Completed	[126]	Gastrointestinal effects	No response
NCT00743288	1, 2	Multiple myeloma	Melphalan	Panobinostat	Completed	[127]	Neutropenia, Thrombocytopenia	7.5% of partial response
NCT01005797	1	Renal cancer, Non-small cell lung cancer, Soft tissue sarcoma	Sorafenib	Panobinostat	Completed			
NCT01336842	1	Solid tumors, Non-small cell lung cancer	Cisplatin, Pemetrexed	Panobinostat	Completed			
NCT01463046	1	Acute myeloid leukemia, Advanced myelodysplastic syndrome	Cytarabine, Daunorubicin	Panobinostat	Completed			
NCT00859222	1, 2	Malignant glioma	Bevacizumab	Panobinostat	Completed	[128,129]	Thrombocytopenia, Hypophosphatemia, Esophageal hemorrhage, Deep venous thrombosis	25% of partial response, 58% of stable disease (Phase1); No significant improvement of 6-month progression-free survival compared with bevacizumab monotherapy (Phase2)
NCT00738751	1	Lung cancer, Head and neck cancer	Erlotinib	Panobinostat	Completed			
NCT00632489	1	Breast cancer	Capecitabine, Lapatinib	Panobinostat	Completed			
NCT00788931	1	HER-2 positive breast cancer, Metastatic breast cancer	Paclitaxel	Panobinostat	Completed			
NCT00946647	1, 2	Myelodysplastic syndromes, Chronic myelomonocytic leukemia, Acute myeloid leukemia	5-Azacytidine	Panobinostat	Completed	[130]	Nausea, Diarrhea, Fatigue, Thrombocytopenia, Vomiting, Constipation	28% of composite complete response
NCT00691938	1, 2	Acute myeloid leukemia, Myelodysplastic syndromes	Decitabine	Panobinostat	Completed	[131]	Fatigue, Febrile neutropenia, Diarrhea, Nausea	5% of complete response/cytogenic complete response
NCT01169636	1, 2	Hodgkin’s lymphoma	Ifosfamide, Carboplatin, Etoposide	Panobinostat	Completed			
NCT01055795	1	Advanced solid tumors	Bevacizumab	Panobinostat	Completed			
NCT00556088	1	Solid tumors	Paclitaxel, Carboplatin, Bevacizumab	Panobinostat	Completed			
NCT00878904	1	Unspecified adult solid tumor	Epirubicin hydrochloride	Panobinostat	Completed	[132]	Thrombocytopenia, Febrile neutropenia, Fatigue	11% of response
NCT02506959	2	Plasma cell leukemia, Plasmacytoma, Recurrent plasma cell myeloma, Refractory plasma cell myeloma	Busulfan, Gemcitabine hydrochloride, Melphalan	Panobinostat	Completed			

## References

[1] McHugh D., Durán I., Gil J. (2025). Senescence as a therapeutic target in cancer and age-related diseases. Nat. Rev. Drug Discov..

[2] Di Micco R., Krizhanovsky V., Baker D., di Fagagna F.D. (2021). Cellular senescence in ageing: From mechanisms to therapeutic opportunities. Nat. Rev. Mol. Cell Biol..

[3] Meguro S., Nakanishi M. (2025). Cellular senescence in the cancer microenvironment. J. Biochem..

[4] Schmitt C.A., Wang B., Demaria M. (2022). Senescence and cancer—Role and therapeutic opportunities. Nat. Rev. Clin. Oncol..

[5] Hayflick L., Moorhead P.S. (1961). The serial cultivation of human diploid cell strains. Exp. Cell Res..

[6] Evangelou K., Lougiakis N., Rizou S.V., Kotsinas A., Kletsas D., Muñoz-Espín D., Kastrinakis N.G., Pouli N., Marakos P., Townsend P. (2017). Robust, universal biomarker assay to detect senescent cells in biological specimens. Aging Cell.

[7] Sharpless N.E., Sherr C.J. (2015). Forging a signature of in vivo senescence. Nat. Rev. Cancer.

[8] Chang J., Wang Y., Shao L., Laberge R.-M., DeMaria M., Campisi J., Janakiraman K., Sharpless N.E., Ding S., Feng W. (2016). Clearance of senescent cells by ABT263 rejuvenates aged hematopoietic stem cells in mice. Nat. Med..

[9] Yosef R., Pilpel N., Tokarsky-Amiel R., Biran A., Ovadya Y., Cohen S., Vadai E., Dassa L., Shahar E., Condiotti R. (2016). Directed elimination of senescent cells by inhibition of BCL-W and BCL-XL. Nat. Commun..

[10] Dimri G.P., Lee X., Basile G., Acosta M., Scott G., Roskelley C., Medrano E.E., Linskens M., Rubelj I., Pereira-Smith O. (1995). A biomarker that identifies senescent human cells in culture and in aging skin in vivo. Proc. Natl. Acad. Sci. USA.

[11] Shah P.P., Donahue G., Otte G.L., Capell B.C., Nelson D.M., Cao K., Aggarwala V., Cruickshanks H.A., Rai T.S., McBryan T. (2013). Lamin B1 depletion in senescent cells triggers large-scale changes in gene expression and the chromatin landscape. Genes Dev..

[12] Gorgoulis V., Adams P.D., Alimonti A., Bennett D.C., Bischof O., Bishop C., Campisi J., Collado M., Evangelou K., Ferbeyre G. (2019). Cellular Senescence: Defining a Path Forward. Cell.

[13] Freund A., Laberge R.-M., Demaria M., Campisi J., Magin T.M. (2012). Lamin B1 loss is a senescence-associated biomarker. Mol. Biol. Cell.

[14] Wang B., Han J., Elisseeff J.H., Demaria M. (2024). The senescence-associated secretory phenotype and its physiological and pathological implications. Nat. Rev. Mol. Cell Biol..

[15] Laberge R.-M., Sun Y., Orjalo A.V., Patil C.K., Freund A., Zhou L., Curran S.C., Davalos A.R., Wilson-Edell K.A., Liu S. (2015). MTOR regulates the pro-tumorigenic senescence-associated secretory phenotype by promoting IL1A translation. Nat. Cell Biol..

[16] Orjalo A.V., Bhaumik D., Gengler B.K., Scott G.K., Campisi J. (2009). Cell surface-bound IL-1alpha is an upstream regulator of the senescence-associated IL-6/IL-8 cytokine network. Proc. Natl. Acad. Sci. USA.

[17] Kuilman T., Michaloglou C., Vredeveld L.C., Douma S., van Doorn R., Desmet C.J., Aarden L.A., Mooi W.J., Peeper D.S. (2008). Oncogene-Induced Senescence Relayed by an Interleukin-Dependent Inflammatory Network. Cell.

[18] Meguro S., Johmura Y., Wang T.-W., Kawakami S., Tanimoto S., Omori S., Okamura Y.T., Hoshi S., Kayama E., Yamaguchi K. (2024). Preexisting senescent fibroblasts in the aged bladder create a tumor-permissive niche through CXCL12 secretion. Nat. Aging.

[19] Takikawa T., Hamada S., Matsumoto R., Tanaka Y., Kataoka F., Sasaki A., Masamune A. (2022). Senescent Human Pancreatic Stellate Cells Secrete CXCR2 Agonist CXCLs to Promote Proliferation and Migration of Human Pancreatic Cancer AsPC-1 and MIAPaCa-2 Cell Lines. Int. J. Mol. Sci..

[20] Cheng N., Kim K.-H., Lau L.F. (2022). Senescent hepatic stellate cells promote liver regeneration through IL-6 and ligands of CXCR2. J. Clin. Investig..

[21] Chambers E.S., Vukmanovic-Stejic M., Shih B.B., Trahair H., Subramanian P., Devine O.P., Glanville J., Gilroy D., Rustin M.H.A., Freeman T.C. (2021). Recruitment of inflammatory monocytes by senescent fibroblasts inhibits antigen-specific tissue immunity during human aging. Nat. Aging.

[22] Kawagoe Y., Kawashima I., Sato Y., Okamoto N., Matsubara K., Kawamura K. (2020). CXCL5-CXCR2 signaling is a senescence-associated secretory phenotype in preimplantation embryos. Aging Cell.

[23] Jin H.J., Lee H.J., Heo J., Lim J., Kim M., Kim M.K., Nam H.Y., Hong G.H., Cho Y.S., Choi S.J. (2016). Senescence-Associated MCP-1 Secretion Is Dependent on a Decline in BMI1 in Human Mesenchymal Stromal Cells. Antioxid. Redox Signal..

[24] Mattia L., Gossiel F., Walsh J.S., Eastell R. (2023). Effect of age and gender on serum growth differentiation factor 15 and its relationship to bone density and bone turnover. Bone Rep..

[25] Guo Y., Ayers J.L., Carter K.T., Wang T., Maden S.K., Edmond D., Polly P.N., Li C., Ulrich C., Yu M. (2019). Senescence-associated tissue microenvironment promotes colon cancer formation through the secretory factor GDF15. Aging Cell.

[26] Cardoso A.L., Fernandes A., Aguilar-Pimentel J.A., de Angelis M.H., Guedes J.R., Brito M.A., Ortolano S., Pani G., Athanasopoulou S., Gonos E.S. (2018). Towards frailty biomarkers: Candidates from genes and pathways regulated in aging and age-related diseases. Ageing Res. Rev..

[27] Hoare M., Ito Y., Kang T.-W., Weekes M., Matheson N., Patten D., Shetty S., Parry A., Menon S., Salama R. (2016). NOTCH1 mediates a switch between two distinct secretomes during senescence. Nat. Cell. Biol..

[28] Acosta J.C., Banito A., Wuestefeld T., Georgilis A., Janich P., Morton J.P., Athineos D., Kang T.-W., Lasitschka F., Andrulis M. (2013). A complex secretory program orchestrated by the inflammasome controls paracrine senescence. Nat. Cell Biol..

[29] Hernandez-Segura A., de Jong T.V., Melov S., Guryev V., Campisi J., DeMaria M. (2017). Unmasking Transcriptional Heterogeneity in Senescent Cells. Curr. Biol..

[30] Braig M., Lee S., Loddenkemper C., Rudolph C., Peters A.H., Schlegelberger B., Stein H., Dörken B., Jenuwein T., Schmitt C.A. (2005). Oncogene-induced senescence as an initial barrier in lymphoma development. Nature.

[31] Liu X.-L., Ding J., Meng L.-H. (2018). Oncogene-induced senescence: A double edged sword in cancer. Acta Pharmacol. Sin..

[32] Courtois-Cox S., Jones S.L., Cichowski K. (2008). Many roads lead to oncogene-induced senescence. Oncogene.

[33] Chan A.S.L., Zhu H., Cassidy L.D., Young A.R.J., Bermejo-Rodriguez C., Janowska A.T., Chen H.-C., Gough S., Oshimori N., Zender L. (2024). Titration of RAS alters senescent state and influences tumour initiation. Nature.

[34] Sturmlechner I., Zhang C., Sine C.C., van Deursen E.-J., Jeganathan K.B., Hamada N., Grasic J., Friedman D., Stutchman J.T., Can I. (2021). p21 produces a bioactive secretome that places stressed cells under immunosurveillance. Science.

[35] Kang T.-W., Yevsa T., Woller N., Hoenicke L., Wuestefeld T., Dauch D., Hohmeyer A., Gereke M., Rudalska R., Potapova A. (2011). Senescence surveillance of pre-malignant hepatocytes limits liver cancer development. Nature.

[36] Xue W., Zender L., Miething C., Dickins R.A., Hernando E., Krizhanovsky V., Cordon-Cardo C., Lowe S.W. (2007). Senescence and tumour clearance is triggered by p53 restoration in murine liver carcinomas. Nature.

[37] Lujambio A., Akkari L., Simon J., Grace D., Tschaharganeh D.F., Bolden J.E., Zhao Z., Thapar V., Joyce J.A., Krizhanovsky V. (2013). Non-Cell-Autonomous Tumor Suppression by p53. Cell.

[38] Ruscetti M., Morris J.P., Mezzadra R., Russell J., Leibold J., Romesser P.B., Simon J., Kulick A., Ho Y.J., Fennell M. (2021). Senescence-Induced Vascular Remodeling Creates Therapeutic Vulnerabilities in Pancreas Cancer. Cell.

[39] Ruscetti M., Leibold J., Bott M.J., Fennell M., Kulick A., Salgado N.R., Chen C.-C., Ho Y.-J., Sanchez-Rivera F.J., Feucht J. (2018). NK cell–mediated cytotoxicity contributes to tumor control by a cytostatic drug combination. Science.

[40] Sternberg C., Raigel M., Limberger T., Trachtová K., Schlederer M., Lindner D., Kodajova P., Yang J., Ziegler R., Kalla J. (2024). Cell-autonomous IL6ST activation suppresses prostate cancer development via STAT3/ARF/p53-driven senescence and confers an immune-active tumor microenvironment. Mol. Cancer.

[41] Colucci M., Zumerle S., Bressan S., Gianfanti F., Troiani M., Valdata A., D’Ambrosio M., Pasquini E., Varesi A., Cogo F. (2024). Retinoic acid receptor activation reprograms senescence response and enhances anti-tumor activity of natural killer cells. Cancer Cell.

[42] Romesser P.B., Lowe S.W. (2023). The Potent and Paradoxical Biology of Cellular Senescence in Cancer. Annu. Rev. Cancer Biol..

[43] Goel S., DeCristo M.J., Watt A.C., BrinJones H., Sceneay J., Li B.B., Khan N., Ubellacker J.M., Xie S., Metzger-Filho O. (2017). CDK4/6 inhibition triggers anti-tumour immunity. Nature.

[44] Chen H.A., Ho Y.J., Mezzadra R., Adrover J.M., Smolkin R., Zhu C., Woess K., Bernstein N., Schmitt G., Fong L. (2023). Senescence Rewires Microenvironment Sensing to Facilitate Antitumor Immunity. Cancer Discov..

[45] Sagiv A., Burton D.G.A., Moshayev Z., Vadai E., Wensveen F., Ben-Dor S., Golani O., Polic B., Krizhanovsky V. (2016). NKG2D ligands mediate immunosurveillance of senescent cells. Aging.

[46] Ruhland M.K., Loza A.J., Capietto A.-H., Luo X., Knolhoff B.L., Flanagan K.C., Belt B.A., Alspach E., Leahy K., Luo J. (2016). Stromal senescence establishes an immunosuppressive microenvironment that drives tumorigenesis. Nat. Commun..

[47] Eggert T., Wolter K., Ji J., Ma C., Yevsa T., Klotz S., Medina-Echeverz J., Longerich T., Forgues M., Reisinger F. (2016). Distinct Functions of Senescence-Associated Immune Responses in Liver Tumor Surveillance and Tumor Progression. Cancer Cell.

[48] Salminen A., Kauppinen A., Kaarniranta K. (2018). Myeloid-derived suppressor cells (MDSC): An important partner in cellular/tissue senescence. Biogerontology.

[49] Toso A., Revandkar A., Di Mitri D., Guccini I., Proietti M., Sarti M., Pinton S., Zhang J., Kalathur M., Civenni G. (2014). Enhancing Chemotherapy Efficacy in Pten-Deficient Prostate Tumors by Activating the Senescence-Associated Antitumor Immunity. Cell Rep..

[50] Ortiz-Montero P., Londoño-Vallejo A., Vernot J.-P. (2017). Senescence-associated IL-6 and IL-8 cytokines induce a self- and cross-reinforced senescence/inflammatory milieu strengthening tumorigenic capabilities in the MCF-7 breast cancer cell line. Cell Commun. Signal..

[51] Luo X., Fu Y., Loza A.J., Murali B., Leahy K.M., Ruhland M.K., Gang M., Su X., Zamani A., Shi Y. (2016). Stromal-Initiated Changes in the Bone Promote Metastatic Niche Development. Cell Rep..

[52] Ancrile B., Lim K.-H., Counter C.M. (2007). Oncogenic Ras-induced secretion of IL6 is required for tumorigenesis. Genes Dev..

[53] Kong P., Yang X., Zhang Y., Dong H., Liu X., Xu X., Zhang X., Shi Y., Hou M., Song B. (2022). Palbociclib Enhances Migration and Invasion of Cancer Cells via Senescence-Associated Secretory Phenotype-Related CCL5 in Non-Small-Cell Lung Cancer. J. Oncol..

[54] Eyman D., Damodarasamy M., Plymate S., Reed M. (2009). CCL5 secreted by senescent aged fibroblasts induces proliferation of prostate epithelial cells and expression of genes that modulate angiogenesis. J. Cell. Physiol..

[55] Liu D., Hornsby P.J. (2007). Senescent Human Fibroblasts Increase the Early Growth of Xenograft Tumors via Matrix Metalloproteinase Secretion. Cancer Res..

[56] Guccini I., Revandkar A., D’AMbrosio M., Colucci M., Pasquini E., Mosole S., Troiani M., Brina D., Sheibani-Tezerji R., Elia A.R. (2021). Senescence Reprogramming by TIMP1 Deficiency Promotes Prostate Cancer Metastasis. Cancer Cell.

[57] Kawaguchi K., Komoda K., Mikawa R., Asai A., Sugimoto M. (2021). Cellular senescence promotes cancer metastasis by enhancing soluble E-cadherin production. iScience.

[58] Kim Y.H., Choi Y.W., Lee J., Soh E.Y., Kim J.-H., Park T.J. (2017). Senescent tumor cells lead the collective invasion in thyroid cancer. Nat. Commun..

[59] Wang T.-W., Nakanishi M. (2025). Immune surveillance of senescence: Potential application to age-related diseases. Trends Cell Biol..

[60] Majewska J., Agrawal A., Mayo A., Roitman L., Chatterjee R., Kralova J.S., Landsberger T., Katzenelenbogen Y., Meir-Salame T., Hagai E. (2024). p16-dependent increase of PD-L1 stability regulates immunosurveillance of senescent cells. Nat. Cell Biol..

[61] Wang T.-W., Johmura Y., Suzuki N., Omori S., Migita T., Yamaguchi K., Hatakeyama S., Yamazaki S., Shimizu E., Imoto S. (2022). Blocking PD-L1–PD-1 improves senescence surveillance and ageing phenotypes. Nature.

[62] Onorati A., Havas A.P., Lin B., Rajagopal J., Sen P., Adams P.D., Dou Z. (2022). Upregulation of PD-L1 in Senescence and Aging. Mol. Cell. Biol..

[63] Reimann M., Schrezenmeier J.F., Richter-Pechanska P., Dolnik A., Hick T.P., Schleich K., Cai X., Fan D.N.Y., Lohneis P., Masswig S. (2021). Adaptive T-cell immunity controls senescence-prone MyD88- or CARD11-mutant B-cell lymphomas. Blood.

[64] Shahbandi A., Chiu F.-Y., Ungerleider N.A., Kvadas R., Mheidly Z., Sun M.J.S., Tian D., Waizman D.A., Anderson A.Y., Machado H.L. (2022). Breast cancer cells survive chemotherapy by activating targetable immune-modulatory programs characterized by PD-L1 or CD80. Nat. Cancer.

[65] Chaib S., López-Domínguez J.A., Lalinde-Gutiérrez M., Prats N., Marin I., Boix O., García-Garijo A., Meyer K., Muñoz M.I., Aguilera M. (2024). The efficacy of chemotherapy is limited by intratumoral senescent cells expressing PD-L2. Nat. Cancer.

[66] Pereira B.I., Devine O.P., Vukmanovic-Stejic M., Chambers E.S., Subramanian P., Patel N., Virasami A., Sebire N.J., Kinsler V., Valdovinos A. (2019). Senescent cells evade immune clearance via HLA-E-mediated NK and CD8+ T cell inhibition. Nat. Commun..

[67] Muñoz D.P., Yannone S.M., Daemen A., Sun Y., Vakar-Lopez F., Kawahara M., Freund A.M., Rodier F., Wu J.D., Desprez P.-Y. (2019). Targetable mechanisms driving immunoevasion of persistent senescent cells link chemotherapy-resistant cancer to aging. J. Clin. Investig..

[68] Sahai E., Astsaturov I., Cukierman E., DeNardo D.G., Egeblad M., Evans R.M., Fearon D., Greten F.R., Hingorani S.R., Hunter T. (2020). A framework for advancing our understanding of cancer-associated fibroblasts. Nat. Rev. Cancer.

[69] Liu L., Huang H., Cheng B., Xie H., Peng W., Cui H., Liang J., Cao M., Yang Y., Chen W. (2025). Revealing the role of cancer-associated fibroblast senescence in prognosis and immune landscape in pancreatic cancer. iScience.

[70] Assouline B., Kahn R., Hodali L., Condiotti R., Engel Y., Elyada E., Mordechai-Heyn T., Pitarresi J.R., Atias D., Steinberg E. (2024). Senescent cancer-associated fibroblasts in pancreatic adenocarcinoma restrict CD8+ T cell activation and limit responsiveness to immunotherapy in mice. Nat. Commun..

[71] Belle J.I., Sen D., Baer J.M., Liu X., Lander V.E., Ye J., Sells B.E., Knolhoff B.L., Faiz A., Kang L.-I. (2024). Senescence Defines a Distinct Subset of Myofibroblasts That Orchestrates Immunosuppression in Pancreatic Cancer. Cancer Discov..

[72] Ye J., Baer J.M., Faget D.V., Morikis V.A., Ren Q., Melam A., Delgado A.P., Luo X., Bagchi S.M., Belle J.I. (2024). Senescent CAFs Mediate Immunosuppression and Drive Breast Cancer Progression. Cancer Discov..

[73] Leone P., Malerba E., Susca N., Favoino E., Perosa F., Brunori G., Prete M., Racanelli V. (2024). Endothelial cells in tumor micro-environment: Insights and perspectives. Front. Immunol..

[74] Ma L., He X., Fu Y., Ge S., Yang Z. (2024). Senescent endothelial cells promote liver metastasis of uveal melanoma in single-cell resolution. J. Transl. Med..

[75] Hwang H.J., Lee Y.R., Kang D., Lee H.C., Seo H.R., Ryu J.K., Kim Y.N., Ko Y.G., Park H.J., Lee J.S. (2020). Endothelial cells under therapy-induced senescence secrete CXCL11, which increases aggressiveness of breast cancer cells. Cancer Lett..

[76] Wang D., Xiao F., Feng Z., Li M., Kong L., Huang L., Wei Y., Li H., Liu F., Zhang H. (2020). Sunitinib facilitates metastatic breast cancer spreading by inducing endothelial cell senescence. Breast Cancer Res..

[77] Wieland E., Rodriguez-Vita J., Liebler S.S., Mogler C., Moll I., Herberich S.E., Espinet E., Herpel E., Menuchin A., Chang-Claude J. (2017). Endothelial Notch1 Activity Facilitates Metastasis. Cancer Cell.

[78] Liu Z., Liang Q., Ren Y., Guo C., Ge X., Wang L., Cheng Q., Luo P., Zhang Y., Han X. (2023). Immunosenescence: Molecular mechanisms and diseases. Signal Transduct. Target. Ther..

[79] Fane M., Weeraratna A.T. (2020). How the ageing microenvironment influences tumour progression. Nat. Rev. Cancer.

[80] Sanoff H.K., Deal A.M., Krishnamurthy J., Torrice C., Dillon P., Sorrentino J., Ibrahim J.G., Jolly T.A., Williams G., Carey L.A. (2014). Effect of Cytotoxic Chemotherapy on Markers of Molecular Age in Patients with Breast Cancer. J. Natl. Cancer Inst..

[81] Amundson S.A., Grace M.B., McLeland C.B., Epperly M.W., Yeager A., Zhan Q., Greenberger J.S., Fornace A.J. (2004). Human in vivo radiation-induced biomarkers: Gene expression changes in radiotherapy patients. Cancer Res..

[82] Schwartz G.K., Shah M.A. (2005). Targeting the Cell Cycle: A New Approach to Cancer Therapy. J. Clin. Oncol..

[83] Zhou B.-B.S., Elledge S.J. (2000). The DNA damage response: Putting checkpoints in perspective. Nature.

[84] Faheem M.M., Seligson N.D., Ahmad S.M., Rasool R.U., Gandhi S.G., Bhagat M., Goswami A. (2020). Convergence of therapy-induced senescence (TIS) and EMT in multistep carcinogenesis: Current opinions and emerging perspectives. Cell Death Discov..

[85] Wang M., Morsbach F., Sander D., Gheorghiu L., Nanda A., Benes C., Kriegs M., Krause M., Dikomey E., Baumann M. (2011). EGF receptor inhibition radiosensitizes NSCLC cells by inducing senescence in cells sustaining DNA double-strand breaks. Cancer Res..

[86] Hasan M.R., Ho S.H.Y., Owen D.A., Tai I.T. (2011). Inhibition of VEGF induces cellular senescence in colorectal cancer cells. Int. J. Cancer.

[87] Amatori S., Bagaloni I., Viti D., Fanelli M. (2011). Premature senescence induced by DNA demethylating agent (Decitabine) as therapeutic option for malignant pleural mesothelioma. Lung Cancer.

[88] Turchick A., Zimmermann A., Chiu L.Y., Dahmen H., Elenbaas B., Zenke F.T., Blaukat A., Vassilev L.T. (2023). Selective Inhibition of ATM-Dependent Double-Strand Break Repair and Checkpoint Control Synergistically Enhances the Efficacy of ATR Inhibitors. Mol. Cancer Ther..

[89] Dobler C., Jost T., Hecht M., Fietkau R., Distel L. (2020). Senescence Induction by Combined Ionizing Radiation and DNA Damage Response Inhibitors in Head and Neck Squamous Cell Carcinoma Cells. Cells.

[90] Vendetti F.P., Lau A., Schamus S., Conrads T.P., O’Connor M.J., Bakkenist C.J. (2015). The orally active and bioavailable ATR kinase inhibitor AZD6738 potentiates the anti-tumor effects of cisplatin to resolve ATM-deficient non-small cell lung cancer in vivo. Oncotarget.

[91] Tesei A., Arienti C., Bossi G., Santi S., De Santis I., Bevilacqua A., Zanoni M., Pignatta S., Cortesi M., Zamagni A. (2021). TP53 drives abscopal effect by secretion of senescence-associated molecular signals in non-small cell lung cancer. J. Exp. Clin. Cancer Res..

[92] Kansara M., Leong H.S., Lin D.M., Popkiss S., Pang P., Garsed D.W., Walkley C.R., Cullinane C., Ellul J., Haynes N.M. (2013). Immune response to RB1-regulated senescence limits radiation-induced osteosarcoma formation. J. Clin. Investig..

[93] Liu Y., Pagacz J., Wolfgeher D.J., Bromerg K.D., Gorman J.V., Kron S.J. (2023). Senescent cancer cell vaccines induce cytotoxic T cell responses targeting primary tumors and disseminated tumor cells. J. Immunother. Cancer.

[94] Meng Y., Efimova E.V., Hamzeh K.W., Darga T.E., Mauceri H.J., Fu Y.-X., Kron S.J., Weichselbaum R.R. (2012). Radiation-inducible Immunotherapy for Cancer: Senescent Tumor Cells as a Cancer Vaccine. Mol. Ther..

[95] Uceda-Castro R., Margarido A.S., Cornet L., Vegna S., Hahn K., Song J.-Y., Putavet D.A., van Geldorp M., Çitirikkaya C.H., de Keizer P.L. (2022). Re-purposing the pro-senescence properties of doxorubicin to introduce immunotherapy in breast cancer brain metastasis. Cell Rep. Med..

[96] Marin I., Boix O., Garcia-Garijo A., Sirois I., Caballe A., Zarzuela E., Ruano I., Attolini C.S., Prats N., Lopez-Dominguez J.A. (2023). Cellular Senescence Is Immunogenic and Promotes Antitumor Immunity. Cancer Discov..

[97] Soriani A., Iannitto M.L., Ricci B., Fionda C., Malgarini G., Morrone S., Peruzzi G., Ricciardi M.R., Petrucci M.T., Cippitelli M. (2014). Reactive Oxygen Species– and DNA Damage Response–Dependent NK Cell Activating Ligand Upregulation Occurs at Transcriptional Levels and Requires the Transcriptional Factor E2F1. J. Immunol..

[98] Hao X., Zhao B., Zhou W., Liu H., Fukumoto T., Gabrilovich D., Zhang R. (2021). Sensitization of ovarian tumor to immune checkpoint blockade by boosting senescence-associated secretory phenotype. iScience.

[99] Hwang H.J., Kang D., Shin J., Jung J., Ko S., Jung K.H., Hong S.-S., Park J.E., Oh M.J., An H.J. (2025). Therapy-induced senescent cancer cells contribute to cancer progression by promoting ribophorin 1-dependent PD-L1 upregulation. Nat. Commun..

[100] Chembukavu S.N., Lindsay A.J. (2024). Therapy-induced senescence in breast cancer: An overview. Explor. Target. Anti-Tumor Ther..

[101] Sun X., Shi B., Zheng H., Min L., Yang J., Li X., Liao X., Huang W., Zhang M., Xu S. (2018). Senescence-associated secretory factors induced by cisplatin in melanoma cells promote non-senescent melanoma cell growth through activation of the ERK1/2-RSK1 pathway. Cell Death Dis..

[102] Jackson J.G., Pant V., Li Q., Chang L.L., Quintás-Cardama A., Garza D., Tavana O., Yang P., Manshouri T., Li Y. (2012). p53-Mediated Senescence Impairs the Apoptotic Response to Chemotherapy and Clinical Outcome in Breast Cancer. Cancer Cell.

[103] Wang L., Lankhorst L., Bernards R. (2022). Exploiting senescence for the treatment of cancer. Nat. Rev. Cancer.

[104] Zhu Y.I., Tchkonia T., Pirtskhalava T., Gower A.C., Ding H., Giorgadze N., Palmer A.K., Ikeno Y., Hubbard G.B., Lenburg M. (2015). The Achilles’ heel of senescent cells: From transcriptome to senolytic drugs. Aging Cell.

[105] Thadathil N., Selvarani R., Mohammed S., Nicklas E.H., Tran A.L., Kamal M., Luo W., Brown J.L., Lawrence M.M., Borowik A.K. (2022). Senolytic treatment reduces cell senescence and necroptosis in Sod1 knockout mice that is associated with reduced inflammation and hepatocellular carcinoma. Aging Cell.

[106] Wang L., Xiong B., Lu W., Cheng Y., Zhu J., Ai G., Zhang X., Liu X., Cheng Z. (2024). Senolytic drugs dasatinib and quercetin combined with Carboplatin or Olaparib reduced the peritoneal and adipose tissue metastasis of ovarian cancer. Biomed. Pharmacother..

[107] Saleh T., Carpenter V.J., Tyutyunyk-Massey L., Murray G., Leverson J.D., Souers A.J., Alotaibi M.R., Faber A.C., Reed J., Harada H. (2020). Clearance of therapy-induced senescent tumor cells by the senolytic ABT-263 via interference with BCL-X(L) -BAX interaction. Mol. Oncol..

[108] Fleury H., Malaquin N., Tu V., Gilbert S., Martinez A., Olivier M.-A., Sauriol S.A., Communal L., Leclerc-Desaulniers K., Carmona E. (2019). Exploiting interconnected synthetic lethal interactions between PARP inhibition and cancer cell reversible senescence. Nat. Commun..

[109] Gonzalez-Gualda E., Paez-Ribes M., Lozano-Torres B., Macias D., Wilson J.R., Gonzalez-Lopez C., Ou H.L., Miron-Barroso S., Zhang Z., Lerida-Viso A. (2020). Galacto-conjugation of Navitoclax as an efficient strategy to increase senolytic specificity and reduce platelet toxicity. Aging Cell.

[110] Wang C., Vegna S., Jin H., Benedict B., Lieftink C., Ramirez C., de Oliveira R.L., Morris B., Gadiot J., Wang W. (2019). Inducing and exploiting vulnerabilities for the treatment of liver cancer. Nature.

[111] Wakita M., Takahashi A., Sano O., Loo T.M., Imai Y., Narukawa M., Iwata H., Matsudaira T., Kawamoto S., Ohtani N. (2020). A BET family protein degrader provokes senolysis by targeting NHEJ and autophagy in senescent cells. Nat. Commun..

[112] Samaraweera L., Adomako A., Rodriguez-Gabin A., McDaid H.M. (2017). A Novel Indication for Panobinostat as a Senolytic Drug in NSCLC and HNSCC. Sci. Rep..

[113] Johmura Y., Yamanaka T., Omori S., Wang T.-W., Sugiura Y., Matsumoto M., Suzuki N., Kumamoto S., Yamaguchi K., Hatakeyama S. (2021). Senolysis by glutaminolysis inhibition ameliorates various age-associated disorders. Science.

[114] Kovacovicova K., Skolnaja M., Heinmaa M., Mistrik M., Pata P., Pata I., Bartek J., Vinciguerra M. (2018). Senolytic Cocktail Dasatinib + Quercetin (D + Q) Does Not Enhance the Efficacy of Senescence-Inducing Chemotherapy in Liver Cancer. Front. Oncol..

[115] Raffaele M., Kovacovicova K., Frohlich J., Re O.L., Giallongo S., Oben J.A., Faldyna M., Leva L., Giannone A.G., Cabibi D. (2021). Mild exacerbation of obesity- and age-dependent liver disease progression by senolytic cocktail dasatinib + quercetin. Cell Commun. Signal..

[116] Hainsworth J.D., Infante J.R., Spigel D.R., Arrowsmith E.R., Boccia R.V., Burris H.A. (2011). A phase II trial of panobinostat, a histone deacetylase inhibitor, in the treatment of patients with refractory metastatic renal cell carcinoma. Cancer Investig..

[117] Rathkopf D.E., Picus J., Hussain A., Ellard S., Chi K.N., Nydam T., Allen-Freda E., Mishra K.K., Porro M.G., Scher H.I. (2013). A phase 2 study of intravenous panobinostat in patients with castration-resistant prostate cancer. Cancer Chemother. Pharmacol..

[118] Murali B., Ren Q., Luo X., Faget D.V., Wang C., Johnson R.M., Gruosso T., Flanagan K.C., Fu Y., Leahy K. (2018). Inhibition of the Stromal p38MAPK/MK2 Pathway Limits Breast Cancer Metastases and Chemotherapy-Induced Bone Loss. Cancer Res..

[119] Jerby-Arnon L., Shah P., Cuoco M.S., Rodman C., Su M.-J., Melms J.C., Leeson R., Kanodia A., Mei S., Lin J.-R. (2018). A Cancer Cell Program Promotes T Cell Exclusion and Resistance to Checkpoint Blockade. Cell.

[120] Amor C., Feucht J., Leibold J., Ho Y.-J., Zhu C., Alonso-Curbelo D., Mansilla-Soto J., Boyer J.A., Li X., Giavridis T. (2020). Senolytic CAR T cells reverse senescence-associated pathologies. Nature.

[121] Tolcher A.W., LoRusso P., Arzt J., Busman T.A., Lian G., Rudersdorf N.S., Vanderwal C.A., Kirschbrown W., Holen K.D., Rosen L.S. (2015). Safety, efficacy, and pharmacokinetics of navitoclax (ABT-263) in combination with erlotinib in patients with advanced solid tumors. Cancer Chemother. Pharmacol..

[122] Vlahovic G., Karantza V., Wang D., Cosgrove D., Rudersdorf N., Yang J., Xiong H., Busman T., Mabry M. (2014). A phase I safety and pharmacokinetic study of ABT-263 in combination with carboplatin/paclitaxel in the treatment of patients with solid tumors. Investig. New Drugs.

[123] Cleary J.M., Lima C.M.S.R., Hurwitz H.I., Montero A.J., Franklin C., Yang J., Graham A., Busman T., Mabry M., Holen K. (2014). A phase I clinical trial of navitoclax, a targeted high-affinity Bcl-2 family inhibitor, in combination with gemcitabine in patients with solid tumors. Investig. New Drugs.

[124] Puglisi M., Molife L.R., de Jonge M.J., Khan K.H., Doorn L.V., Forster M.D., Blanco M., Gutierrez M., Franklin C., Busman T. (2021). A Phase I study of the safety, pharmacokinetics and efficacy of navitoclax plus docetaxel in patients with advanced solid tumors. Future Oncol..

[125] Pietanza M.C., Rudin C.M. (2012). Novel Therapeutic Approaches for Small Cell Lung Cancer: The Future has Arrived. Curr. Probl. Cancer.

[126] Goldberg J., Sulis M.L., Bender J., Jeha S., Gardner R., Pollard J., Aquino V., Laetsch T., Winick N., Fu C. (2020). A phase I study of panobinostat in children with relapsed and refractory hematologic malignancies. Pediatr. Hematol. Oncol..

[127] Berenson J.R., Hilger J.D., Yellin O., Boccia R.V., Matous J., Dressler K., Ghazal H.H., Jamshed S., Kingsley E.C., Harb W.A. (2014). A phase 1/2 study of oral panobinostat combined with melphalan for patients with relapsed or refractory multiple myeloma. Ann. Hematol..

[128] Drappatz J., Lee E.Q., Hammond S., Grimm S.A., Norden A.D., Beroukhim R., Gerard M., Schiff D., Chi A.S., Batchelor T.T. (2012). Phase I study of panobinostat in combination with bevacizumab for recurrent high-grade glioma. J. Neuro-Oncol..

[129] Lee E.Q., Reardon D.A., Schiff D., Drappatz J., Muzikansky A., Grimm S.A., Norden A.D., Nayak L., Beroukhim R., Rinne M.L. (2015). Phase II study of panobinostat in combination with bevacizumab for recurrent glioblastoma and anaplastic glioma. Neuro-Oncology.

[130] Garcia-Manero G., Sekeres M.A., Egyed M., Breccia M., Graux C., Cavenagh J.D., Salman H., Illes A., Fenaux P., DeAngelo D.J. (2017). A phase 1b/2b multicenter study of oral panobinostat plus azacitidine in adults with MDS, CMML or AML with ≤30% blasts. Leukemia.

[131] Uy G.L., Duncavage E.J., Chang G.S., Jacoby M.A., Miller C.A., Shao J., Heath S., Elliott K., Reineck T., Fulton R.S. (2017). Dynamic changes in the clonal structure of MDS and AML in response to epigenetic therapy. Leukemia.

[132] Thomas S., Aggarwal R., Jahan T., Ryan C., Troung T., Cripps A.M., Raha P., Thurn K.T., Chen S., Grabowsky J.A. (2016). A phase I trial of panobinostat and epirubicin in solid tumors with a dose expansion in patients with sarcoma. Ann. Oncol..

